# Efficacy of Intraoperative Anterior Segment Optical Coherence Tomography during Descemet's Stripping Automated Endothelial Keratoplasty

**DOI:** 10.1155/2014/562062

**Published:** 2014-02-02

**Authors:** Akio Miyakoshi, Hironori Ozaki, Mitsuya Otsuka, Atsushi Hayashi

**Affiliations:** Department of Ophthalmology, Graduate School of Medicine and Pharmaceutical Sciences, University of Toyama, 2630 Sugitani, Toyama 930-0194, Japan

## Abstract

*Purpose*. To examine the graft-host interface during Descemet's stripping automated endothelial keratoplasty (DSAEK) surgery with optical coherence tomography (OCT). *Design*. Prospective, interventional case series. *Patients and Methods*. Eight patients who underwent a DSAEK were included. A handheld OCT was used intraoperatively to examine the presence of interface fluid between the host cornea and the graft. *Results*. In 3 patients, no interface fluid was detected between the host cornea and the graft after the graft was attached by air injection. In 4 patients, interface fluid was detected after the graft was attached by air injection. The remaining interface fluid was drained through corneal stab incisions. One patient required a second surgery because the first surgery failed due to persistence of the interface fluid. All patients showed a complete attachment of the graft at one month after the DSAEK surgery. *Conclusion*. A handheld OCT is useful to detect the interface fluid between the host cornea and the graft during a DSAEK.

## 1. Introduction

Penetrating keratoplasty has mainly been performed for the treatment of bullous keratopathy and other diseases with corneal endothelial dysfunction. Recently, DSAEK (Descemet's stripping automated endothelial keratoplasty) has been performed as a primary surgical treatment for such diseases [1–3]. And although DSAEK is more advantageous for the recovery of visual acuity than penetrating keratoplasty, its complications include postoperative graft detachment and graft dislocation [1–7]. The rates of these complications vary between 4% and 50% [1–7]. Several studies have been conducted to identify a means of preventing the graft-host interface from being detached or being dislocated intraoperatively or postoperatively [8–10]. Two of these studies reported that anterior segment optical coherence tomography (OCT) is useful to assess the status of the graft-host interface [8, 10].

In this study, therefore, we prospectively examined the graft-host interface during a DSAEK with a handheld OCT.

## 2. Materials and Methods

We included 8 eyes of 8 consecutive patients (3 men and 5 women) who underwent DSAEK for the treatment of bullous keratopathy at Toyama University Hospital between October 2010 and February 2012. The study protocol was approved by the Institutional Review Board of the University of Toyama, and the procedures used conformed to the tenets of the Declaration of Helsinki. Each patient provided written informed consent after hearing a detailed explanation of the study protocol.

In this study, the cornea and the anterior segment were examined with a handheld OCT during the DSAEK surgery and followed up for more than one month after the surgery. The mean age at the time of surgery was 63.0 ± 18.3 years (mean ± standard deviation). We examined the relationship between the intraoperative conditions of the graft-host interface and the postoperative conditions.

Eight consecutive eyes with vision loss resulting from corneal edema from bullous keratopathy were selected for the study. Only patients with pseudophakia or aphakia were included. Eyes with significant anterior stromal scarring were excluded.

The patients had been diagnosed with pseudophakic bullous keratopathy (PBK) (2 eyes, 25%), Fuchs corneal endothelial dystrophy (2 eyes, 25%), laser iridotomy-induced bullous keratopathy (LIBK) (2 eyes, 25%), aphakic bullous keratopathy (1 eye, 13%), and pseudoexfoliation syndrome (1 eye, 13%). Each patient was treated with DSAEK surgery alone, and all surgeries were performed by the same surgeon (Atsushi Hayashi).

## 3. OCT

**Figure 1:**
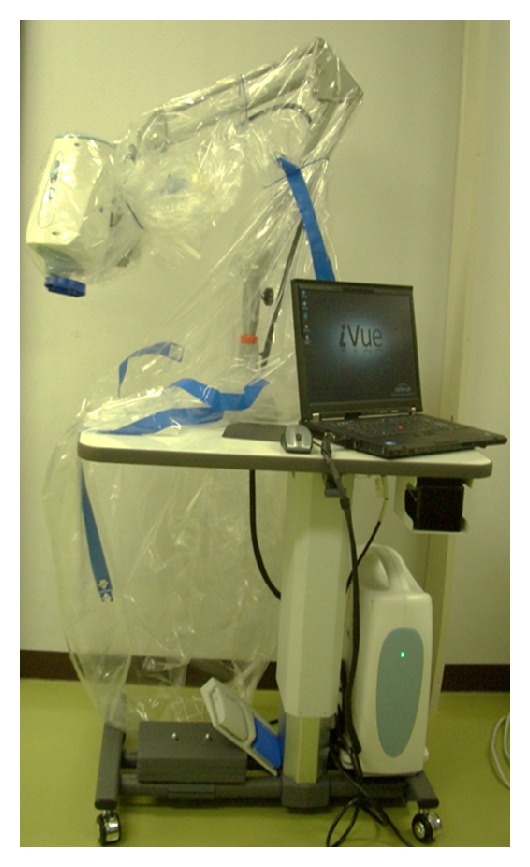
Photograph of the anterior segment spectral-domain optical coherence tomography device used intraoperatively in this series.

We used an iVue-100 (ver.1.7) as a handheld OCT and performed anterior segment OCT scans while the patient was in a supine position during the surgery (Figure 1). A corneal anterior module was attached, and the eyes were scanned using a corneal pachymetry scan pattern. Eight radial scans of 6 mm diameter were obtained at a speed of 1024 A-scans/second.

## 4. Surgical Techniques

We used sclerocorneal tissues provided by the Toyama Eye Bank for surgical transplantation. During the surgery, the sclerocorneal tissue was mounted on an artificial anterior chamber, tightened completely, and dissected with a microkeratome equipped with a 350 *μ*m cutter head. The precut donor tissue was trephined with a 7.5 mm diameter corneal punch, and a donor endothelial graft was obtained. Then, sub-Tenon's anesthesia of 2% Lidocaine (1.5–2 mL) was administered. An inferior peripheral iridectomy was performed with a vitreous cutter. We inserted the endothelial graft according to the following procedure. A DSAEK Busin Glide Spatula was used to insert the graft into the anterior chamber from a 5 mm temporal sclerocorneal incision, while the anterior chamber depth was maintained with an infusion of balanced salt solution (BSS) by an anterior chamber maintainer. Only in the eyes with Fuchs corneal endothelial dystrophy, the Descemet's membrane was removed with a reverse Sinskey hook. A 10–0 nylon suture was used to suture the sclerocorneal wound, and the air was injected into the anterior chamber using a 30-gauge needle to attach the graft onto the inner surface of the host cornea. After the graft was attached, the anterior segment was examined with a handheld OCT (iVue-100; Optovue Inc., Fremont, CA). When interface fluid was detected between the host cornea and the graft, it was drained through corneal stab incisions, and then the OCT scans were repeated. When the graft attachment was confirmed by OCT scans, the intraocular pressure (IOP) was set within the normal range, and the surgery was finished. The patients were asked to remain in a supine position for 3 hours after surgery.

## 5. Results

**Figure 2:**
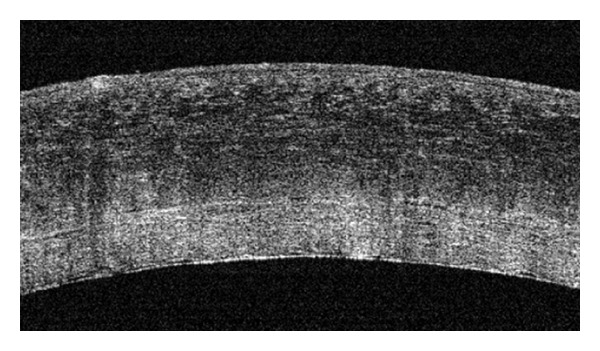
An OCT image of the eye of patient 1 after the first air injection. No interface fluid was detected.

All 8 patients successfully underwent DSAEK without complications. We removed the host Descemet's membrane during the surgery in the 2 patients with Fuchs dystrophy and did not remove the host Descemet's membrane in the other 6 patients. A summary of the results of each case is given in Table 1. During DSAEK in patient 1, no interface fluid was detected between the host cornea and the graft by a handheld OCT after the injection of air (Figure 2). We did not encounter a postoperative graft dislocation the day after surgery in this patient.

**Figure 3:**
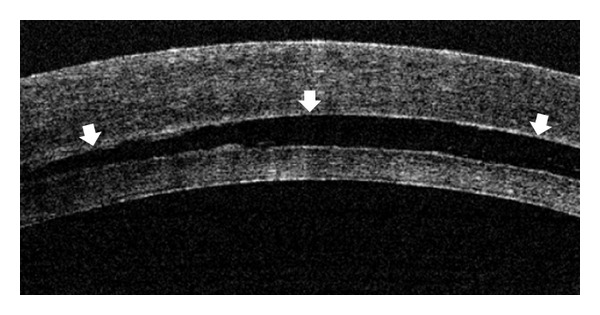
An OCT image of the eye of patient 2 at the end of the first DSEAK surgery. Interface fluid was observed between the host cornea and the donor graft (arrows).

During DSAEK in patient 2 with Fuchs corneal endothelial dystrophy, viscoelastic material was used to maintain the anterior chamber during removal of the Descemet's membrane with a reverse hook. The viscoelastic material was washed out from the anterior chamber after the removal of the Descemet's membrane, and the graft was inserted into the anterior chamber. The graft was attached to the inner surface of the cornea by an injection of air. The handheld OCT revealed interface fluid between the host cornea and the graft in this patient, and we tried to drain this fluid through corneal stab incisions. However, a small amount of interface fluid remained at the end of the surgery (Figure 3). A few days after the surgery, a double anterior chamber was observed, and at 7 days after the surgery graft dislocation was noted inferiorly. We performed a reoperation and the graft was again attached onto the inner surface of the cornea without any interface fluid by the OCT examination. The graft was attached after the second surgery. For the remaining six patients, we performed the DSAEK surgery without any viscoelastic material.

**Figure 4:**
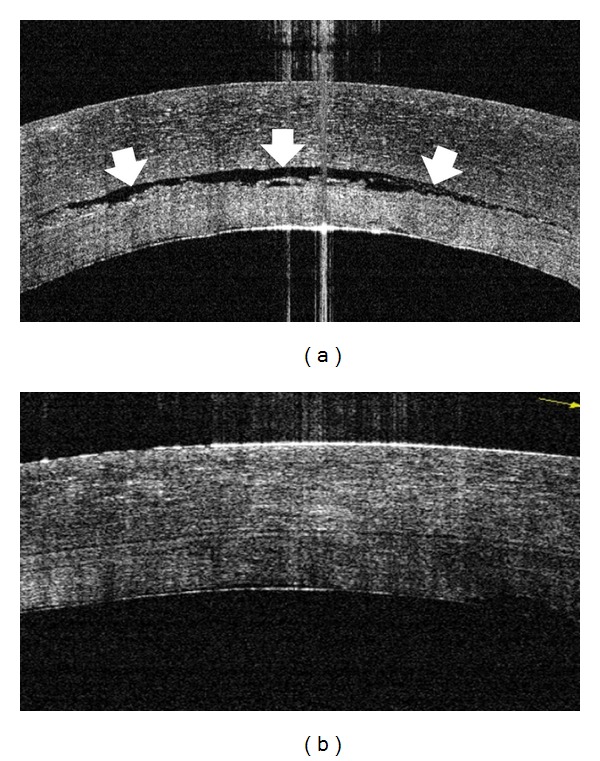
OCT images of the operated eye of patient 4 during the DSEAK surgery. (a) Interface fluid was observed between the donor and the host cornea after the first air injection (arrows). (b) After the fluid was drained through corneal stub incisions, no interface fluid was detected by the OCT.

In patients 3 and 5, OCT examination during the surgery revealed no interface fluid. Because patients 4, 6, 7, and 8 showed small amounts of interface fluid by the OCT examination after the injection of air, we drained the interface fluid through corneal stab incisions. We then confirmed that there was no detectable interface fluid by OCT examination and finished the surgery (Figure 4).

The grafts were attached at one month after the DSAEK in all patients.

## 6. Discussion

DSAEK is superior to penetrating keratoplasty in terms of the recovery of visual acuity, the survival rates of corneal endothelial cells, and the rejection rates and does not cause strong corneal astigmatism [3, 11, 12].

In patient 2, the first surgery failed due to the remaining interface fluid detected by the intraoperative OCT. Because we used a viscoelastic material to maintain a working space for removal of the Descemet's membrane from the host cornea during the surgery, a small amount of the viscoelastic material may have remained on the inner surface of the host cornea and disturbed the graft attachment. It was difficult to distinguish whether the remaining fluid was balanced salt solution or viscoelastic material by the OCT examination. Hence, it might be important to use only BSS for the irrigation of the anterior chamber during the DSAEK surgery and to routinely examine for the presence of interface fluid during the DSAEK by means of OCT examination.

In this series, interface fluid was detected in 5 of the 8 eyes (62.5%) after the graft was attached by air injection. When even some amount of interface fluid is detected, it should be removed through corneal stub incisions for the success of the DSAEK surgery. Hyams et al. reported that, in a case where the interface space was present for more than 2 weeks, the growth of fibrous tissue was triggered and corneal opacity remained [13]. A slit lamp attached to an operating microscope is commonly used to detect interface fluid between the host cornea and the graft. However, Tanawska and Wylegala reported that interface space was detected in 32% of their cases when an anterior segment OCT was used for the examination, whereas it was detected only in 16% of their cases when slit lamp was used because the examination was performed one day after DSAEK [9]. Because the OCT detects interface fluid more accurately than a slit lamp attached to an operating microscope, it would be important to check the interface fluid intraoperatively with a handheld OCT and to finish the DSAEK without any interface fluid. Hence, an anterior segment OCT is effective to assess the interface space during the surgery.

The anterior segment OCT used in this study appears to help prevent postoperative graft dislocation and graft detachment, since it allows intraoperative confirmation of the presence of interface fluid. Moreover, it reduces retreatment procedures such as postoperative rebubbling and repositioning. Price and Price Jr. compared 17 cases retreated postoperatively with 246 cases without retreatment and reported that the endothelial cells were significantly decreased in the retreated cases [14]. This result suggested that intraoperative removal of the interface fluid detected by an anterior segment OCT may help to protect the endothelial cells of the graft.

## 7. Conclusion

A handheld OCT is useful to detect the interface fluid between the host cornea and the graft during a DSAEK.

## Figures and Tables

**Table 1:** Summary of Descemet's stripping automated endothelial keratoplasty cases.

Patient no.	Age	Sex	Reason for DSAEK	Intraoperative OCT	Postoperative OCT	Graft attachment after the first surgery
1	40	M	PBK	No space	No space	Success
2	55	F	Fuchs	Space	Space	Fail
3	81	M	Fuchs	No space	No space	Success
4	83	F	PBK	Space	No space	Success
5	70	F	LIBK	No space	No space	Success
6	68	F	PE	Space	No space	Success
7	73	F	LIBK	Space	No space	Success
8	34	M	ABK	Space	No space	Success

PBK: pseudophakic bullous keratopathy, LIBK: laser iridotomy bullous keratopathy, PE: pseudoexfoliation, and ABK: aphakic bullous keratopathy.
